# Myofascial Release for the Treatment of Tension-Type, Cervicogenic Headache or Migraine: A Systematic Review and Meta-Analysis

**DOI:** 10.1155/2024/2042069

**Published:** 2024-03-31

**Authors:** Zhoupeng Lu, Hui Zou, Peng Zhao, Jialin Wang, Ruirui Wang

**Affiliations:** ^1^Sports Rehabilitation Research Center, China Institute of Sport Science, Beijing, China; ^2^College of Sports Medicine and Physical Therapy, Beijing Sport University, Beijing, China

## Abstract

**Objective:**

To assess the effectiveness of myofascial release (MFR) techniques on the intensity of headache pain and associated disability in patients with tension-type headache (TTH), cervicogenic headache (CGH), or migraine.

**Design:**

A systematic review and meta-analysis.

**Methods:**

Eight databases were searched on September 15, 2023, including PubMed, Scopus, Web of Science, CINAHL, Cochrane Library, Embase, CNKI, and Wanfang Database. The risk of bias was evaluated utilizing the Cochrane Risk of Bias 2 (RoB 2) tool.

**Results:**

Pooled results showed that MFR intervention significantly reduces pain intensity [SMD = −2.01, 95% CI (−2.98, −1.03), *I*^2^ = 90%, *P* < 0.001] and improves disability [SMD = −1.3, 95% CI (−1.82, −0.79), *I*^2^ = 74%, *P* < 0.001]. Subgroup analysis based on the type of headache revealed significant reductions in pain intensity for CGH [SMD = −2.01, 95% CI (−2.73, −1.29), *I*^2^ = 63%, *P* < 0.001], TTH [SMD = −0.86, 95% CI (−1.52, −0.20), *I*^2^ = 50%, *P*=0.01] and migraine [SMD = −6.52, 95% CI (−8.15, −4.89), *P* < 0.001] and in disability for CGH [SMD = −1.45, 95% CI (−2.07, −0.83), *I*^2^ = 0%, *P* < 0.001]; TTH [SMD = −0.98, 95% CI (−1.32, −0.65), *I*^2^ = 0%, *P* < 0.001] but not migraine [SMD = −2.44, 95% CI (−6.04, 1.16), *I*^2^ = 97%, *P*=0.18].

**Conclusion:**

The meta-analysis results indicate that MFR intervention can significantly alleviate pain and disability in TTH and CGH. For migraine, however, the results were inconsistent, and there was only moderate quality evidence of disability improvement for TTH and CGH. In contrast, the quality of other evidence was low or very low.

## 1. Introduction

Headache is one of the most common neurological symptoms, yet the extent and scale of headaches have been consistently underestimated, and they still lack full recognition and treatment worldwide [1]. Currently, the global prevalence of headaches is 47%, and the proportion of people who have experienced headaches at least once in their lifetime is even higher, reaching 66%. This exerts a significant impact and burden on both individuals and society [2]. The International Headache Society classifies headaches into primary and secondary categories [3]. Primary headaches are those without a clear cause and signs or test results indicating other diseases or abnormalities. Tension-type headaches (TTH) and migraine are representative examples of primary headaches [3]. The prevalence of TTH is 38%, and the proportion of individuals who have experienced TTH at least once in their lifetime is 46% [2]. The prevalence of migraine is 10%, and the proportion of individuals who have experienced migraines at least once in their lifetime is 14% [2].

Secondary headaches are those caused by other diseases or factors, and they are a symptom rather than a distinct medical condition [3]. Cervicogenic headache (CGH) is a prevalent form of secondary headache. The term “cervicogenic headache” was initially introduced in 1983, describing a syndrome primarily defined by persistent head pain, either unilateral or bilateral, resulting from mechanical or functional issues in the cervical spine or cervical soft tissues [4]. It is estimated to affect 2.5% of the general population and is observed in 17.8% of individuals who experience frequent headaches [5]. Nevertheless, there remains ongoing debate within clinical practice concerning this definition [6]. Diagnosing CGH in a clinical setting is challenging due to the overlapping clinical features outlined by the Cervicogenic Headache International Study Group (CHISG). These features include unilateral headache, along with symptoms like nausea, photophobia, phonophobia, and neck pain, which are shared with other headache types such as TTH and migraine [7]. As per Bogduk, the singular defining criterion for CGH is head pain originating from the neck [8]. Furthermore, some literature suggests that the intensity of headache pain in CGH patients may be due to sustained poor head/neck posture or excessive digital pressure on trigger points in the neck or cervical region [9].

Although pharmaceutical treatments are common, they come with significant side effects [10, 11]. The Canadian Headache Society and the European Federation of Neurological Societies have indicated that nonpharmacological manual therapies such as massage and spinal manipulation appear to be a practical approach for alleviating headaches [12, 13]. Additionally, a survey showed that manual therapy is commonly employed to alleviate symptoms of tension-type headaches [14]. In recent years, systematic reviews and meta-analyses have investigated the effectiveness of manual therapy for headaches, revealing optimistic results [15–17]. Myofascial release (MFR), a form of manual therapy, has been widely used in clinical practice. Previous studies have found active myofascial trigger points frequently in TTH and migraine, triggering these headaches upon palpation [18]. Therefore, releasing these myofascial trigger points may be an effective headache treatment. Existing trials have shown that direct and indirect MFRs are effective for TTH [19]. Nevertheless, there is presently an absence of a systematic review and meta-analysis regarding the efficacy of MFR for various headache types. This systematic review aims to evaluate the effectiveness of MFR in reducing the intensity of headache pain and alleviating associated disability in individuals diagnosed with CGH, TTH, or migraine.

## 2. Methods

This systematic review and meta-analysis followed the guidelines outlined in the Preferred Reporting Items for Systematic Reviews and Meta-Analyses (PRISMA) and the Cochrane Handbook for systematic reviews of interventions [20, 21]. Ethical approval was unnecessary for this study, as all analyses were conducted using previously published data. The systematic review is registered at https://www.crd.york.ac.uk/prospero with the identifier CRD42023472041, and we did not prepare a protocol.

### 2.1. Selection Criteria

#### 2.1.1. Study Types

This study exclusively incorporated randomized controlled trials (RCTs). The included literature was confined to English and Chinese.

#### 2.1.2. Patients

The study involved adult participants diagnosed with TTH, CGH, and migraine while excluding adolescents (those under 18 years of age).

#### 2.1.3. Intervention

MFR, such as suboccipital muscle inhibition. There were no constraints on the specific method of MFR, the intervention frequency, or the intervention duration. In cases where combined interventions were employed in the study, all participants in the MFR and the control groups underwent identical combined interventions before being deemed eligible for the study.

#### 2.1.4. Outcomes

The change in pain scores, from the baseline assessment to the latest available follow-up, was assessed utilizing several scales, including the numerical rating scale (NRS) and visual analog scale (VAS). Higher scores on these scales indicate a higher level of pain intensity. The alteration in disability scores, from the initial assessment to the most recent follow-up, was evaluated through various scales, including the neck disability index (NDI), headache impact test (HIT-6), head disability index (HDI), and migraine disability assessment (MIDAS). Higher scores on these scales indicate a greater degree of disability.

### 2.2. Search Strategy

Search strategy: eight databases were searched on September 15, 2023, which included PubMed, Scopus, Web of Science, CINAHL, Cochrane Library, Embase, CNKI, and Wanfang Database. The search results were also updated on October 22, 2023. Two researchers (LZP and ZH) conducted independent searches following the designated search strategy. Upon completion of the search process, an initial screening was conducted by evaluating the titles and abstracts to determine their eligibility for inclusion. Full texts of relevant literature were then reviewed, and further selection was based on inclusion criteria. Any discrepancies will be addressed through deliberation, and if a unanimous agreement cannot be achieved, a third reviewer (WJL) will make the ultimate decision. The specific screening process is detailed in Supplementary Materials.

### 2.3. Data Extraction

Two researchers (LZP and ZH) will conduct a full-text review of the included articles and extract data using a data extraction table. The data extraction table has author, year, disease, treatment type, number of participants in the analysis/randomization, treatment frequency, outcome measures, outcomes, and adverse events. Any discrepancies will be addressed through deliberation, and if a unanimous agreement cannot be achieved, a third reviewer (WJL) will make the ultimate decision.

### 2.4. Assessment of Risk of Bias in Included Studies

Two researchers (LZP and ZH) will employ the Cochrane Risk of Bias 2 (RoB 2) tool for assessing the risk of bias [22]. If the result of the ROB-2 assessment indicates a high risk of bias, the article will be excluded from the analysis. Any discrepancies will be addressed through deliberation, and if a unanimous agreement cannot be achieved, a third reviewer (WJL) will make the ultimate decision.

### 2.5. Rating Quality of Evidence

Two researchers (LZP and ZH) will evaluate the quality of evidence concerning myofascial release in the context of CGH, TTH, and migraine using the GRADE (Grading of Recommendations, Assessment, Development, and Evaluation) tool. According to GRADE guidelines, an assessment will be performed for each outcome measure, with categorizations of high, moderate, low, or very low quality [23].

### 2.6. Data Analysis

When multiple comparisons exist within the same study, as per the Cochrane Handbook, splitting the control group into two groups or combining the intervention groups is recommended to prevent duplicate counting [21]. The total effect sizes based on mean difference and 95% confidence interval were calculated by using the means (standard deviation) of continuous outcome variables after treatment. In cases where studies used different scales to evaluate the same outcome, the standardized mean difference (SMD) was calculated. SMD was used to standardize the results and remove any influence of dimension and measurement methods. The meta-analysis will use Review Manager (RevMan) software, version 5.4. The results of statistical data are shown using forest maps. A random or fixed effects model was employed, and a 95% confidence interval was computed. The heterogeneity tests were analyzed using *I*^2^ and chi-square tests. If *I*^2^ < 50% (*p* ≥ 0.1), signifying no statistically significant difference in heterogeneity, the fixed effects model was utilized for statistical analysis. If *I*^2^ ≥ 50% (*p* < 0.1) indicates a statistically significant difference in heterogeneity, a random effects model was employed for statistical analysis. Funnel plots were used to evaluate the presence of publication bias in the included studies.

## 3. Results

**Figure 1:**
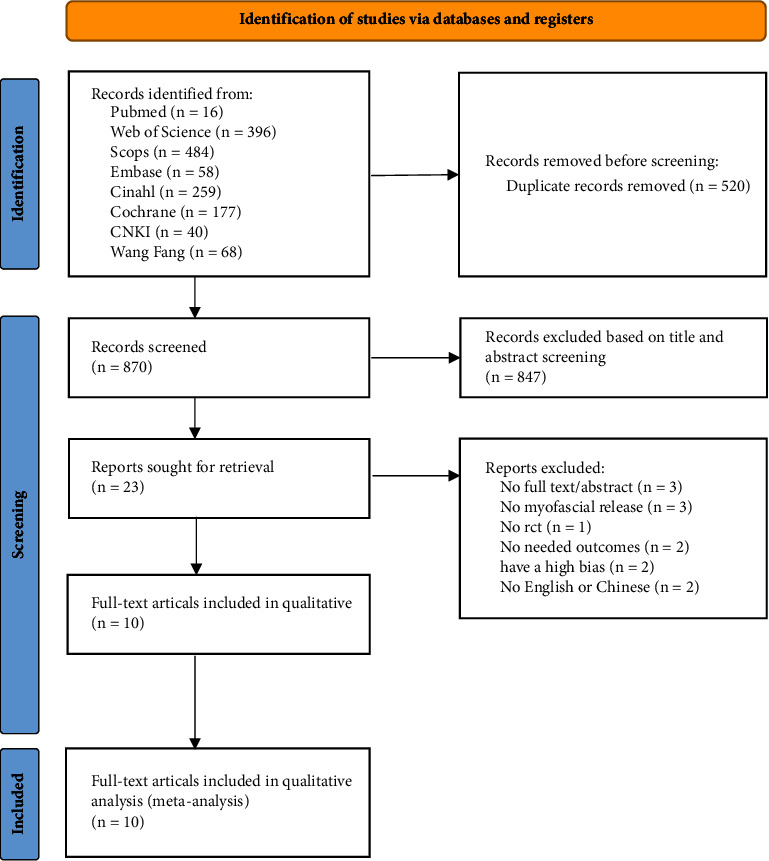
The process of literature retrieval.

Through searches in eight databases, a total of 1390 articles were retrieved. After eliminating duplicate articles through a review process, 847 articles remained. After reviewing titles and abstracts, 23 articles remained. Among these, three articles did not have full texts or abstracts, one was not an RCT, three lacked MFR intervention, two did not have the required outcome measures, and two were excluded due to the high risk of bias. An additional two articles were excluded due to language reasons (Supplementary Materials). Ultimately, ten articles were included in the meta-analysis [24–33]. A detailed literature search process is shown in Figure 1.

### 3.1. General Study Characteristics

This review summarizes the fundamental characteristics of the ten RCTs included. We included ten studies, five focused on TTH, three on CGH, and two on migraine. The included studies encompassed five Asian countries (China [24], India [25], Pakistan [26], Iran [28], Turkey [30], and South Korea [29, 32]) and two European and American countries (Spain [27] and the United States [33]). In the ten studies, 432 headache patients were enrolled, 232 receiving MFR intervention, while 200 were assigned to the control groups. Due to the absence of age and gender data in the two articles [25, 26], it was impossible to calculate the gender distribution and average age. The sample size ranged from 22 to 124, and the range of sample losses was 0 to 12, with a major focus on three studies [29, 30, 32]. Moreover, all studies did not report adverse events (Table 1).

### 3.2. Intervention Characteristics and Outcome Measures

This review summarizes the interventions, intervention time, frequency, and outcome measures used in ten RCTs. In the experimental group, the intervention methods included MFR, MFR + a physiotherapy program, MFR + exercise, MFR + mobilization + drug therapy, and MFR + stretching. The control group interventions included no intervention, placebo control, drug therapy, exercise, physiotherapy program, and SNAG technique + physiotherapy program. Among them, seven RCTs included pain outcome measures: four used VAS [24, 25, 30, 33], and three used NRS [26, 28, 31]. In nine RCTs, disability outcomes were measured: six used NDI [25, 26, 28, 30, 31, 33], three used HIT-6 [27, 30, 32], one used HDI [28], and one used MIDAS [27]. One RCT [27] used both MIDAS and HIT-6 to assess disability, while one RCT [30] used HIT-6 and NDI separately to evaluate disability (Table 1).

### 3.3. Risk of Bias and Quality Assessment

**Figure 2:**
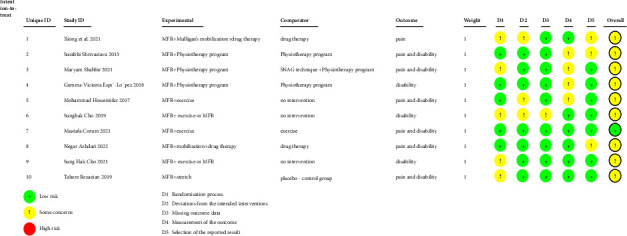
Risk of bias summary for randomized controlled trials.

As high-risk bias studies have already been excluded, the overall bias risk for all studies is either low or of some concern. Regarding the bias during the randomization process, five studies had low bias, three had some concern in terms of deviation from the predefined intervention, one had some concern regarding missing outcome data, four had some concern concerning outcome measurement bias, and three had some concern regarding selective reporting of results. Among all the included articles, only one [30] had an overall low risk of bias. The bias risks determined by ROB-2 assessment are presented in Figure 2.

### 3.4. Quality of Evidence

In this review, the GRADE system was utilized to assess the quality of evidence for each outcome. The results indicated moderate-quality evidence suggesting that MFR intervention benefits disability in TTH and CGH. In contrast, very low-quality evidence suggests an improvement in disability for migraine. Low-quality evidence suggests an improvement in pain for CGH patients with MFR intervention, whereas pain improvement in TTH and migraine is supported by very low-quality evidence (Table 2).

### 3.5. Effects of Interventions

#### 3.5.1. Pain Intensity

**Figure 3:**
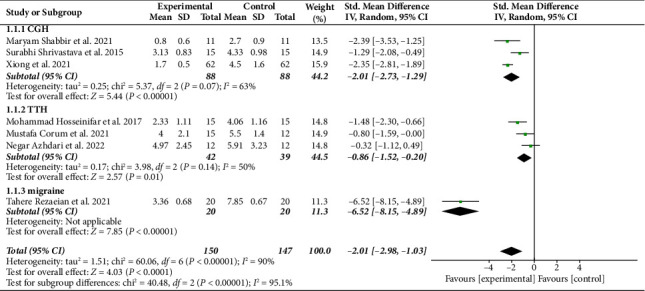
Forest plot of pain intensity.

Pain intensity assessments were conducted in seven studies involving 297 headache patients, with four studies using VAS and three using NRS. Pooled results show that MFR intervention significantly reduces pain intensity [SMD = −2.01, 95% CI (−2.98, −1.03), *I*^2^ = 90%, *P* < 0.001]. Subgroup analysis based on the type of headache revealed significant reductions in pain intensity for CGH [SMD = −2.01, 95% CI (−2.73, −1.29), *I*^2^ = 63%, *P* < 0.001], TTH [SMD = −0.86, 95% CI (−1.52, −0.20), *I*^2^ = 50%, *P*=0.01], and migraine [SMD = −6.52, 95% CI (−8.15, −4.89), *P* < 0.001] (Figure 3).

#### 3.5.2. Disability

**Figure 4:**
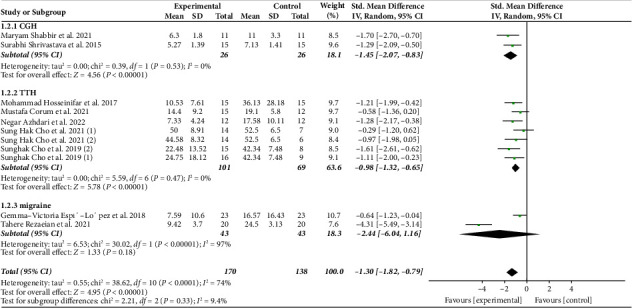
Forest plot of disability.

Nine studies involving a total of 308 headache patients assessed disability using four different scales: NDI, HIT-6, HDI, and MIDAS. Pooled results show that MFR intervention significantly reduces disability levels [SMD = −1.3, 95% CI (−1.82, −0.79), *I*^2^ = 74%, *P* < 0.001]. Subgroup analysis based on the type of headache revealed significant reductions in disability for CGH [SMD = −1.45, 95% CI (−2.07, −0.83), *I*^2^ = 0%, *P* < 0.001] and TTH [SMD = −0.98, 95% CI (−1.32, −0.65), *I*^2^ = 0%, *P* < 0.001] but not migraine [SMD = −2.44, 95% CI (−6.04, 1.16), *I*^2^ = 97%, *P*=0.18] (Figure 4).

#### 3.5.3. Publication Bias

**Figure 5:**
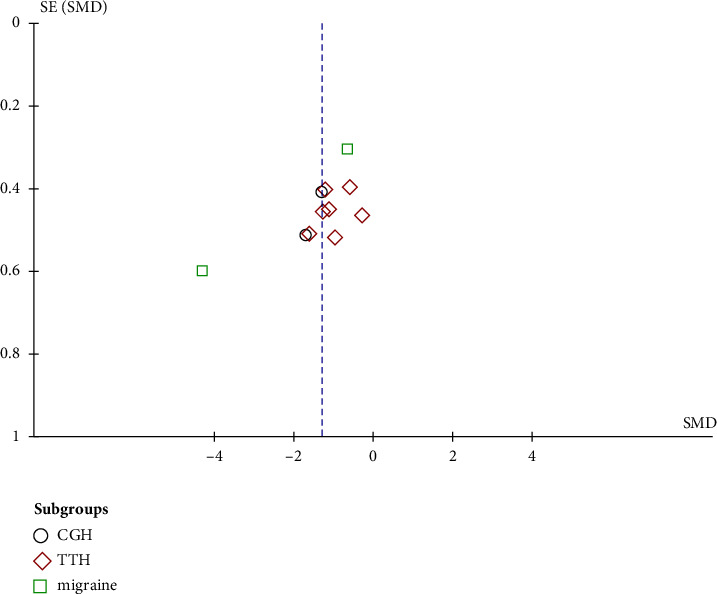
Funnel plot.

Funnel plots were employed in this review to evaluate publication bias in most of the included studies. The symmetry of the funnel plot suggested an absence of publication bias (Figure 5).

## 4. Discussion

The primary objective of this systematic review and meta-analysis is to examine the efficacy of MFR intervention compared to other interventions for patients suffering from headaches. Although the overall results show evidence of pain and disability improvement in headache patients with MFR intervention, the evidence for pain relief in migraine patients from MFR intervention comes from only one article. Only the proof of disability improvement in CGH and TTH is of moderate quality, while the rest of the outcome measures are of low or very low quality. Furthermore, only three articles in the study conducted follow-ups [30, 31, 33], with follow-up times ranging from three days to three months, making it impossible to pool and analyze the data. Since none of the RCTs in this review reported adverse events, making any statements regarding safety and compliance is currently impossible.

As far as our knowledge extends, this represents the inaugural systematic review and meta-analysis appraising the efficacy of MFR intervention in patients with headaches. In recent years, there have been increasing meta-analyses of the effectiveness of MFR intervention in various types of diseases, and the results from these meta-analyses are similar to our findings [34–38]. The studies indicate a positive role of myofascial release in alleviating symptoms related to low back pain [34, 35], fibromyalgia [36], neck pain [37], and orthopedic conditions [38], such as pain relief, improvement in sleep quality, and disability levels.

Headaches may have multiple triggering factors, such as medication overuse [11], alcohol [39, 40], obstructive sleep apnea [41], and sleep disorders [42], but at the same time, myofascial mechanisms are likely closely linked to headaches. Myofascia is a connective tissue that envelops muscles and nerves and can sense pain and pressure [43]. When myofascial is stimulated or inflamed, it may trigger a reflex response, leading to vasoconstriction or dilation, affecting blood flow and oxygen supply to the head, resulting in headaches [44]. Currently, research has found that the number of active trigger points in headache patients has significantly increased, and myofascial trigger points are likely one of the causes of head and neck pain, not just a concomitant phenomenon [45]. MFR is achieved by applying sustained low-load, long-duration stretching forces to restore the length, flexibility, and health of myofascia [46]. Moreover, in vitro studies have indicated that MFR can potentially lower the production of inflammatory cytokines [47]. Therefore, MFR is likely an effective intervention for alleviating headaches. The outcomes of this meta-analysis suggest that MFR may be an effective approach for mitigating pain and reducing disability in patients with headaches; however, this conclusion should be viewed as preliminary. This may be due to the relatively low methodological quality of the included RCTs, with only one article having an overall low risk of bias. This led to a downgrade in the quality of evidence for all outcomes. Additionally, the number of incorporated articles and the sample size were relatively small, potentially influencing intergroup comparisons. Moreover, only a few articles conducted follow-ups, and the follow-up periods were relatively short, limiting the observation of the long-term effects of MFR intervention. Furthermore, the intervention methods and durations varied among the included studies, and other treatments were administered alongside MFR. Different forms and durations of MFR intervention may yield diverse results, contributing to the observed high heterogeneity. Lastly, except for two studies [24, 31] that explicitly used drug therapy as a control group, the remaining studies did not report the use of drugs, and the participants' use of drugs may be a source of heterogeneity.

### 4.1. Implications for Further Research

Future RCTs should utilize more robust study designs and adhere to the CONSORT guidelines to minimize the risk of bias [48]. Additionally, in clinical studies on MFR, future research should extend the duration and frequency of follow-up assessments to evaluate both short-term and long-term effects of MFR intervention on patients with headaches. Furthermore, detailed records of procedural operations, duration, and applied force should be maintained during MFR intervention for future studies to facilitate standardization.

### 4.2. Clinical Implications

Despite its preliminary nature, the conclusion suggests that MFR is a straightforward and efficient method for alleviating pain and disability in headache patients, making it a viable option in clinical practice alongside drug therapy.

## 5. Conclusion

MFR intervention can significantly alleviate pain and disability in TTH and CGH. For migraine, however, the results were inconsistent, and there was only moderate quality evidence of disability improvement for TTH and CGH. In contrast, the quality of other evidence was low or very low. Due to the limited number of included studies and the low quality of evidence, future research should incorporate more rigorously designed RCTs to validate these conclusions.

## Figures and Tables

**Table 1:** General studies' characteristics and intervention characteristics and outcome measures.

References	Country	Disease type	Participant (male/female)			Age (years)		Interventions and time		Intervention length, frequency, and duration		Needed outcomes
			Sum	EG	CG	EG	CG	EG	CG	EG	CG	
Xiong et al. [24]	China	CGH	124	62 (27/35)	62 (29/33)	41.6 ± 7.8	38.7 ± 6.3	MFR + Mulligan's mobilization + drug therapy	Drug therapy	The control group was treated with the same basic drugs + for 10 days, and fascia manual release combined with dynamic joint loosening was treated once a day	Celecoxib gum 200 mg once a day, eperisone hydrochloride tablet 50 mg 3 times a day, compound danshen tablet 960 mg 3 times a day. The duration of treatment was 10 days	Pain (VAS)

Shrivastava et al. [25]	India	CGH	30	15 (unspecified)	15 (unspecified)			MFR + physiotherapy program	Physiotherapy program	Five times a week for six weeks	Five times a week for six weeks	Pain (VAS), disability (NDI)

Shabbir et al. [26]	Pakistan	CGH	22	11 (3/8)	11 (4/7)	30.63 ± 4.90	33.18 ± 3.62	MFR + physiotherapy program	SNAG technique + Physiotherapy program	Three times a week for six weeks	Three times a week for six weeks	Pain (NRS) disability (NDI)

Espí-López et al. [27]	Spain	Migraine	46	23 (1/22)	23 (7/16)	33.9 ± 11.5	33.9 ± 11.5	MFR + physiotherapy program (30 min)	Physiotherapy program (30 min)	8 weeks, 4 sessions (once every 15 days)	8 weeks, 4 sessions (once every 15 days)	Disability (HIT-6,MIDAS)

Hosseinifar et al. [28]	Iran	TTH	30	15 (female)	15 (female)	25.06 ± 7.64	29.33 ± 11.63	MFR + exercise (45 minutes)	No intervention	Four times a week for three weeks		Pain (NRS) disability (HDI)

Cho et al. [29]	Korea	TTH	60	16 (5/11)	17 (5/12)	33.23 ± 8.74	38.07 ± 10.94	MFR (15 minutes)	No intervention	Twice a week, 4 weeks/twice a week, 4 weeks + 3 extra training sessions at home		Disability (NDI)
				15 (4/11)		35.56 ± 8.57		MFR (15 minutes) + exercise				

Corum et al. [30]	Turkey	TTH	30	15 (4/11)	12 (4/8)	30.7 ± 8.0	32.5 ± 6.5	MFR (10 minutes) + exercise	Exercise	MFR for twice a week and exercise for three times a week, 4 weeks	Three times a week, 4 weeks	Pain (VAS), disability (NDI,HIT-6)
Azhdari et al. [31]	Iran	TTH	24	12 (5/7)	12 (4/8)	43.16	41.41	MFR + mobilization + drug therapy (30 min)	Drug therapy	Three therapy sessions for one week	Nortriptyline 50 qsh and valporae Na 200 mg BID	Pain (NRS) disability (NDI)

Cho et al. [32]	Korea	TTH	45	14 (3/11)	13 (3/10)	36.60 ± 8.33	39.07 ± 10.99	MFR (15 minutes)	No intervention	Twice a week, 4 weeks/twice a week, 4 weeks + 3 extra training sessions at home		Disability (HIT-6)
				14 (4/10)		36.00 ± 8.47		MFR (15 minutes) + exercise				

Mosallanezhad et al. [33]	USA	Migraine	40	20 (12/8)	20 (12/8)	40.4 ± 11.2	37.45 ± 8.9	MFR + stretch (20 minutes)	Placebo-control	Three times a week for two weeks	Three times a week for two weeks	Pain (VAS) disability (NDI)

CGH, cervicogenic headache; TTH, tension-type headache; EG, experiment group; CG, control group; MFR, myofascial release; VAS, visual analog scale; NDI, neck disability index; HIT-6, Headache Impact Test; MIDAS, migraine disability assessment; HDI, head disability index; NRS, numerical rating scale.

**Table 2:** Quality of evidence.

Headache type	Outcome	No. of studies	Limitations	Inconsistency	Indirectness	Imprecision	Publication bias	Evidence quality
CGH	Pain	3	Serious	Serious	Not serious	Not serious	Undetected	Low
	Disability	2	Serious	Not serious	Not serious	Serious	Undetected	Moderate

TTH	Pain	3	Serious	Serious	Not serious	Serious	Undetected	Very low
	Disability	5	Serious	Not serious	Not serious	Not serious	Undetected	Moderate

Migraine	Pain	1	n/a	Serious	Not serious	Serious	n/a	Very low
	Disability	2	Serious	Serious	Not serious	Serious	Undetected	Very low

CGH, cervicogenic headache; TTH, tension-type headache; n/a = because only one study was included in the meta-analysis.

## Data Availability

Some or all of the data produced or examined during this study have been incorporated into this published article or the data repositories referenced.

## Abbreviations

TTH: Tension-type headache
CGH: Cervicogenic headache
MFR: Myofascial release
VAS: Visual analog scale
MIDAS: Migraine disability assessment
HIT-6: Headache Impact Test
HDI: Head disability index
NDI: Neck disability index
NRS: Numerical rating Scale
PRISMA: Preferred reporting items for systematic reviews and meta-analysis
RCT: Randomized controlled trial.

## Additional Points

*Key Findings*. MFR may be an effective intervention for alleviating headaches.

## Supplementary Materials

Supplementary Materials (1) Search strategy. (2) After reading the full text, the study was excluded and the reasons for the exclusion.
