# Supplementary material for: Myofascial Release for the Treatment of Tension-Type, Cervicogenic Headache or Migraine: A Systematic Review and Meta-Analysis
Source: Pain Res Manag. 2024 Mar 31;2024:2042069. doi: 10.1155/2024/2042069 (PMC10999287; doi:10.1155/2024/2042069)
Supplement: Supplementary Materials — (1) Search strategy. (2) After reading the full text, the study was excluded and the reasons for the exclusion. [file 2042069.f1.docx]

**Supplementary materials**

1. **Search strategy**

**Pubmed**：

((((((((("myofascial release therapy"[Mesh]) ) OR (Myofascial Release Therapies[Title/Abstract])) OR (Therapy, Myofascial Release[Title/Abstract])) OR (Myofascial Release[Title/Abstract])) OR (Myofascial Release Treatment*[Title/Abstract])) OR (Treatment, Myofascial Release[Title/Abstract])) OR (Myofascial Treatment*[Title/Abstract])) OR (Treatment, Myofascial[Title/Abstract])) AND (((((((((((((((((((((((((((((((((((((((("Tension-Type Headache"[Mesh]) OR ("Post-Traumatic Headache"[Mesh])) OR ("Migraine Disorders"[Mesh])) undefined (Headache*, Tension-Type[Title/Abstract])) OR (Tension Type Headache*[Title/Abstract])) OR (Idiopathic Headache*[Title/Abstract])) OR (Headache*, Idiopathic[Title/Abstract])) OR (Stress Headache*[Title/Abstract])) OR (Headache*, Stress[Title/Abstract])) OR (Tension Headache*[Title/Abstract])) OR (Headache*, Tension[Title/Abstract])) OR (Psychogenic Headache*[Title/Abstract])) OR (Headache*, Psychogenic[Title/Abstract])) OR (Tension-Vascular Headache*[Title/Abstract])) OR (Headache*, Tension-Vascular[Title/Abstract])) OR (Tension Vascular Headache*[Title/Abstract])) OR (Headache*, Post-Traumatic[Title/Abstract])) OR (Post Traumatic Headache*[Title/Abstract])) OR (Cervicogenic Headache*[Title/Abstract])) OR (Headache*, Cervicogenic[Title/Abstract])) OR (Disorder*, Migraine[Title/Abstract])) OR (Migraine Disorder[Title/Abstract])) OR (Migraine*[Title/Abstract])) OR (Migraine Headache*[Title/Abstract])) OR (Migraine Headache*[Title/Abstract])) OR (Headache*, Migraine[Title/Abstract])) OR (Acute Confusional Migraine*[Title/Abstract])) OR (Migraine*, Acute Confusional[Title/Abstract])) OR (Status Migrainosus[Title/Abstract])) OR (Hemicrania Migraine*[Title/Abstract])) OR (Migraine*, Hemicrania[Title/Abstract])) OR (Migraine Variant*[Title/Abstract])) OR (Variant*, Migraine[Title/Abstract])) OR (Sick Headache*[Title/Abstract])) OR (Headache*, Sick[Title/Abstract])) OR (Abdominal Migraine*[Title/Abstract])) OR (Migraine*, Abdominal[Title/Abstract])) OR (Cervical Migraine Syndrome*[Title/Abstract])) OR (Migraine Syndrome*, Cervical[Title/Abstract])) OR (chronic tension headache[Title/Abstract])) 16

**Web of science：**

1: ((TS=(headache*) AND TS=(tension-type)) OR (TS=(headache*) AND TS=(“tension type”))

OR (TS=(idiopathic) AND TS=(headache*)) OR (TS=(stress) AND TS=(headache*)) OR

(TS=(tension) AND TS=(headache*)) OR (TS=(pressure) AND TS=(headache*)) OR TS=(

“pressure headache”) OR TS=(“pressure headaches”) OR (TS=(psychogenic) AND

TS=(headache*)) OR (TS=(tension-vascular) AND TS=(headache*)) OR (TS=(“tension

vascular”) AND TS=(headache*)) OR TS=(“tension vascular headache”) OR TS=(“tension

vascular headaches”) OR (TS=(post-traumatic) AND TS=(headache*)) OR (TS=(“post

traumatic”) AND TS=(headache*)) OR (TS=(cervicogenic) AND TS=(headache*)) OR

(TS=(migraine) AND TS=(disorder*)) OR TS=(migraine*) OR (TS=(migraine) AND

TS=(headache*)) OR (TS=( “acute confusional”) AND TS=(migraine*)) OR TS=(“status

migrainosus”) OR TS=(hemicrania) OR TS=(“status hemicranicus”) OR TS=(“familial

migraine”) OR (TS=(hemicrania) AND TS=(migraine*)) OR (TS=(migraine) AND

TS=(variant*)) OR (TS=(sick) AND TS=(headache*)) OR (TS=(abdominal) AND

TS=(migraine*)) OR TS=(“secondary headache disorders”) OR (TS=(“headache disorders”)

AND TS=(secondary)) OR (TS=(“headache disorder”) AND TS=(secondary)) OR

TS=(“secondary headache disorder”) OR TS=(“secondary headache”) OR TS=(“secondary

headaches”) OR (TS=(cervical) AND TS=(“migraine syndromes”)) OR TS=(“chronic tension

headache”)) and Preprint Citation Index (Exclude – Database) Date Run: Thu Oct 22 2023 19:01:33 GMT+0800 Results: 90996

2: myofascial release therapy (Topic) OR Myofascial Release Therapies (Topic) OR Therapy, Myofascial Release (Topic) OR Myofascial Release (Topic) OR Myofascial Release Treatment* (Topic) OR Treatment, Myofascial Release (Topic) OR Myofascial Treatment* (Topic) OR Treatment, Myofascial (Topic) and Preprint Citation Index (Exclude – Database) Date Run: Thu Oct 26 2023 19:01:33 GMT+0800 Results: 5144

3: #1 AND #2 and Preprint Citation Index (Exclude – Database) Date Run: Thu Oct 22 2023 19:01:34 GMT+0800 Results: 396

Scopus:

( ( TITLE-ABS-KEY ( myofascial AND release AND therapy ) OR TITLE-ABS-KEY ( myofascial release AND therapies ) OR TITLE-ABS-KEY ( therapy, myofascial release ) OR TITLE-ABS-KEY ( myofascial release ) OR TITLE-ABS-KEY ( myofascial release AND treatment* ) OR TITLE-ABS-KEY ( treatment, myofascial release ) OR TITLE-ABS-KEY ( myofascial treatment* ) OR TITLE-ABS-KEY ( treatment, myofascial ) ) ) AND ( ( TITLE-ABS-KEY ( headache*, AND tension-type ) OR TITLE-ABS-KEY ( tension type headache* ) OR TITLE-ABS-KEY ( idiopathic headache* ) OR TITLE-ABS-KEY ( headache*, AND idiopathic ) OR TITLE-ABS-KEY ( stress headache* ) OR TITLE-ABS-KEY ( headache*, AND stress ) OR TITLE-ABS-KEY ( tension headache* ) OR TITLE-ABS-KEY ( headache*, AND tension ) OR TITLE-ABS-KEY ( psychogenic headache* ) OR TITLE-ABS-KEY ( headache*, AND psychogenic ) OR TITLE-ABS-KEY ( tension-vascular headache* ) OR TITLE-ABS-KEY ( headache*, AND tension-vascular ) OR TITLE-ABS-KEY ( tension AND vascular headache* ) OR TITLE-ABS-KEY ( headache*, AND post-traumatic ) OR TITLE-ABS-KEY ( post AND traumatic AND headache* ) OR TITLE-ABS-KEY ( cervicogenic AND headache* ) OR TITLE-ABS-KEY ( headache*, AND cervicogenic ) OR TITLE-ABS-KEY ( disorder*, migraine ) OR TITLE-ABS-KEY ( migraine disorder ) OR TITLE-ABS-KEY ( migraine* ) OR TITLE-ABS-KEY ( migraine headache* ) OR TITLE-ABS-KEY ( headache*, migraine ) OR TITLE-ABS-KEY ( acute AND confusional migraine* ) OR TITLE-ABS-KEY ( migraine*, AND acute AND confusional ) OR TITLE-ABS-KEY ( status AND migrainosus ) OR TITLE-ABS-KEY ( hemicrania migraine* ) OR TITLE-ABS-KEY ( migraine*, AND hemicrania ) OR TITLE-ABS-KEY ( migraine variant* ) OR TITLE-ABS-KEY ( variant*, migraine ) OR TITLE-ABS-KEY ( sick AND headache* ) OR TITLE-ABS-KEY ( headache*, AND sick ) OR TITLE-ABS-KEY ( abdominal migraine* ) OR TITLE-ABS-KEY ( migraine*, AND abdominal ) OR TITLE-ABS-KEY ( cervical migraine syndrome* ) OR TITLE-ABS-KEY ( migraine syndrome*, AND cervical ) OR TITLE-ABS-KEY ( chronic AND tension AND headache ) ) ) 484

**CINAHL：**

TX ( myofascial release therapy or Myofascial Release Therapies or Therapy, Myofascial Release or Myofascial Release or Myofascial Release Treatment* or Treatment, Myofascial Release or Myofascial Treatment* or Treatment, Myofascial ) AND TX ( Headache*, Tension-Type or Tension Type Headache* or Idiopathic Headache* or Headache*, Idiopathic or Stress Headache* or Headache*, Stress or Tension Headache* or Headache*, Tension or Psychogenic Headache* or Headache*, Psychogenic or Tension-Vascular Headache* or Headache*, Tension-Vascular or Tension Vascular Headache* or Headache*, Post-Traumatic or Post Traumatic Headache* or Cervicogenic Headache* or Headache*, Cervicogenic or Disorder*, Migraine or Migraine Disorder or Migraine* or Migraine Headache* or Headache*, Migraine or Acute Confusional Migraine* or Migraine*, Acute Confusional or Status Migrainosus or Hemicrania Migraine* or Migraine*, Hemicrania or Migraine Variant* or Variant*, Migraine or Sick Headache* or Headache*, Sick or Abdominal Migraine* or Migraine*, Abdominal or Cervical Migraine Syndrome*or Migraine Syndrome*, Cervical or chronic tension headache ) 259

**Embase：**

#1. 'tension type headache' OR 'headache*, 70,602 22 Oct 2023

tension-type':ab,ti OR

'tension type headache*':ab,ti OR

'idiopathic headache*':ab,ti OR 'headache*,

idiopathic':ab,ti OR 'stress headache*':ab,ti OR

'headache*, stress':ab,ti OR 'tension

headache':ab,ti OR 'headache*, tension':ab,ti OR

'psychogenic headache*':ab,ti OR 'headache*,

psychogenic':ab,ti OR

'tension-vascular headache*':ab,ti OR 'headache*,

tension-vascular':ab,ti OR 'tension

vascular headache*':ab,ti OR 'headache*,

post-traumatic':ab,ti OR 'post traumatic

headache*':ab,ti OR 'cervicogenic

headache*':ab,ti OR 'headache*,

cervicogenic':ab,ti OR

'disorder*, migraine':ab,ti OR

'migraine disorder':ab,ti OR migraine*:ab,ti OR

'migraine headache*':ab,ti OR

'headache*, migraine':ab,ti OR 'acute

confusional migraine*':ab,ti OR 'migraine*, acute

confusional':ab,ti OR 'status

migrainosus':ab,ti OR

'hemicrania migraine*':ab,ti OR 'migraine*,

hemicrania':ab,ti OR 'migraine variant*':ab,ti OR

'variant*, migraine':ab,ti OR 'sick

headache*':ab,ti OR 'headache*, sick':ab,ti OR

'abdominal migraine*':ab,ti OR 'migraine*,

abdominal':ab,ti OR

'cervical migraine syndrome*':ab,ti OR

'migraine syndrome*, cervical':ab,ti OR 'chronic

tension headache':ab,ti

#2. 'myofascial release therapy'/exp OR 'myofascial 1,137 22 Oct 2023

release therapy' OR (myofascial AND

('release'/exp OR release) AND ('therapy'/exp OR

therapy)) OR 'myofascial release therapies':ab,ti

OR 'therapy, myofascial release':ab,ti OR

'myofascial release':ab,ti OR 'myofascial release

treatment*':ab,ti OR

'treatment, myofascial release':ab,ti OR

'myofascial treatment*':ab,ti OR

'treatment, myofascial':ab,ti

#3. #1 AND #2 58 22 Oct 2023

**Cochrane：**

#1 (myofascial release):ti,ab,kw OR (Myofascial Release Therapies):ti,ab,kw OR (Therapy, Myofascial Release):ti,ab,kw OR (Myofascial Release Treatment*):ti,ab,kw OR (Treatment, Myofascial Release):ti,ab,kw OR (Myofascial Treatment*):ti,ab,kw OR (Treatment, Myofascial):ti,ab,kw 2773

#2 (((“Headache”) OR “Migraine Disorders” OR “Tension-Type Headache” OR “Tension-Type Headache” OR “Post-Traumatic Headache” OR “Cervicogenic Headache”)) 40317

#3 #1 AND #2 177

**CNKI：**

(Myofascial release + myofascial release + myofascial treatment + myofascial release + fascial treatment + fascial release) AND (Migraine + tension headache + cervical headache) 40

**Wangfang**

(Migraine OR cervical headache OR tension headache) AND (myofascial release OR myofascial release OR myofascial treatment OR myofascial release) 68

| **2. After reading the full text, the study was excluded and the reasons for exclusion** | |
| --- | --- |
| Exclusion criteria and excluded articles (n = 13) | |
| cochrance study design(n=2) | Trial ID=NCT05383365^1^  Trial ID=IRCT20190221042794N1^2^ |
| Not a rct(n=1) | Wang 2014 ^3^ |
| Lacked MFR intervention(n=3) | Cheng 2016^4^  George Georgoudis 2018^5^  Espi-Lopez 2022^6^ |
| no needed outcomes(n=2) | Nagrale A.V. 2009^7^  M.S. Ajimsha 2011^8^ |
| high risk of bias(n=2) | Seongyeol Kim 2022^9^  Venkata Naveen Kumar V 2020^10^ |
| Language(n=2) | Ankita Sharma 2022^11^  GYEONG, LEE HWA 2021^12^ |
| just abstract(n=1) | Amir Massoud Arab 2018^13^ |
